# Paddy-upland rotation for sustainable agriculture with regards to diverse soil microbial community

**DOI:** 10.1038/s41598-018-26181-2

**Published:** 2018-05-22

**Authors:** Ping-Fu Hou, Chia-Hung Chien, Yi-Fan Chiang-Hsieh, Kuan-Chieh Tseng, Chi-Nga Chow, Hao-Jen Huang, Wen-Chi Chang

**Affiliations:** 1 Kaohsiung District Agricultural Research and Extension Station, Pingtung County, 90846 Taiwan; 2 Institute of Tropical Plant Sciences, National Cheng Kung University, Tainan, 70101 Taiwan; 3 Department of Life Sciences, National Cheng Kung University, Tainan, 70101 Taiwan

## Abstract

Diverse soil microbial community is determinant for sustainable agriculture. Rich microbial diversity has presumably improved soil health for economic crops to grow. In this work, the benefits of paddy-upland rotation on soil microbial diversity and specific microbes are thus intensively explored. The microbiome from multiple factor experiment (three fertilizations coupled with two rotation systems) were investigated by novel enrichment and co-occurrence analysis in a field well maintained for 25 years. Using next-generation sequencing technique, we firstly present explicit evidence that different rotation systems rather than fertilizations mightily governed the soil microbiome. Paddy-upland rotation (R1) obviously increase more microbial diversity than upland rotation (R2) whether organic (OF), chemical (CF) or integrated fertilizers (IF) were concomitantly applied. Besides, the specific bacterial composition dominated in OF soil is more similar to that of R1 than to CF, suggesting that paddy-upland rotation might be the best option for sustainable agriculture if chemical fertilizer is still required. Interestingly, the pot bioassay verified clearly the novel analysis prediction, illustrating that greater microbial diversity and specific microbial composition correlated significantly with disease resistance. This finding highlights the eminence of paddy-upland rotation in promoting microbial diversity and specific microbial compositions, preserving soil health for sustainable agriculture.

## Introduction

Sustainable agriculture heavily relies on healthy soil with superior characteristics, such as divergent species and an abundant eco-friendly microbial population^1–3^. Soil microbes have been known playing significant roles in nutrient cycling, decomposition of organic matter, improving fertility and crop health^4,5^. However, application of excessive chemical fertilizer dropped soil organic carbon to 1.9–2.8%, half of the agriculture soil less than 2% in recent years^6^. Numerous studies have showed that extreme soil environments limit microbial diversity^7,8^, whereas neutral soil and the use of organic fertilizers can enrich microbial variety^9,10^. Improving the microbial diversity through rotation coupled with fertilizers might thus offer a more economic, efficient and ecology-friendly strategy for greater microbial diversity and soil health.

Paddy-upland farming system is commonly applied in tropical agriculture. Although it is believed that the application of paddy-upland rotation can alter either soil physical or chemical properties, improve soil quality and fertility, and optimize crop yields from the experience of the ancients, fully understanding the characteristics of soil in paddy-upland cropping systems is necessary^11,12^. Besides, the effect of microbial diversity on crop diseases have not been made a clear conclusion, especially lack of field survey. Before the development of next generation sequencing (NGS) technology, it has been reported that applying organic fertilizer increased microbial diversity^13^, and might decrease the occurrence of southern blight^14^. Therefore, the relationship between microbial diversity and crop diseases on these soils is worth exploring.

Recently, NGS technology was widely applied to explore the soil microbial composition^8,15^. However, the recent methods usually restrict the comparison of microbiome on a specific taxonomic status in previous studies^16^ Therefore, we used a novel hypergenometric approach (i.e. enrichment analysis) to comprehensively and objectively compare the composition of soil microbial under all taxonomic status in different circumstances. Besides, co-occurrence method was also firstly utilized to evaluate the operational taxonomic units (OTUs) expression among the same rotation or fertilization group. We were therefore prompted to investigate and evaluate the soil microbiological characteristic upon three fertilizations (OF, IF, CF) under two rotation systems (R1, R2) to open a new avenue for sustainable agriculture worldwide.

## Results

### Higher essential elements accumulation in R1 and OF soil

**Figure 1:**
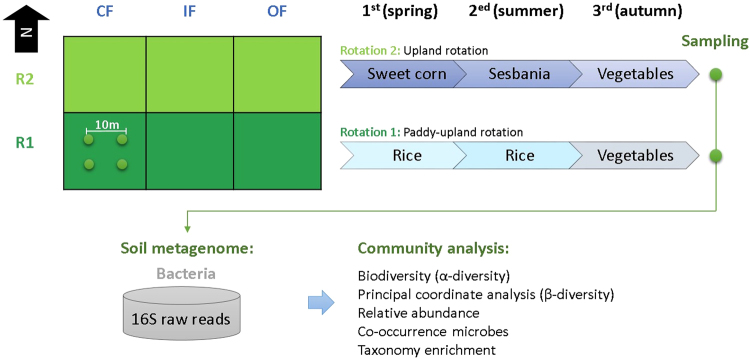
The overview of experimental design and workflow. Six long-term farming methods consist of two crop rotations (Rotation 1 and 2) treated with three kinds of fertilizers: organic (OF), chemical (CF), and integrated (IF, half OF/half CF). The paddy-upland rotation of *rice−rice−vegetables* was implemented in Rotation 1 (R1), whereas Rotation 2 (R2) applied the continuous upland cropping of *sweet corn−sesbania−vegetables*. Four soil samples (10 meters apart) were collected from each farmland. The community analysis was completed after performing the metagenomic sequencing of 16S rRNA libraries.

The full factorial experiment with two variables, rotations (R1 and R2) and fertilizers (OF, CF, and IF), has been carried out in six long-term farming approaches (e.g. R1.25.CF, R1.25.IF, R1.25.OF, R2.25.CF, R2.25.IF, and R2.25.OF) since 1988 (>25 years) (Fig. 1). Some essential mineral elements (i.e. organic matter, K, Ca and Mg) accumulate significantly higher in the order of OF, IF and CF (Fig. S2). In addition, the content of some elements (e.g. organic matter, K, Ca, Mg, Fe, Mn, Cu, Zn and Na) exists significantly different between two rotation systems under the same fertilization regime. Moreover, organic matter substantially accumulated in R1OF (5.59 g kg^−1^) rather than R2OF (4.52 g kg^−1^)) (Table S1) as well. These findings reveal that the R1OF cultivation increase more carbon sequestration than R2OF in soil, and providing best soil nutrients for plant growth so far.

### Rotation system determines microbial composition and biodiversity

**Figure 2:**
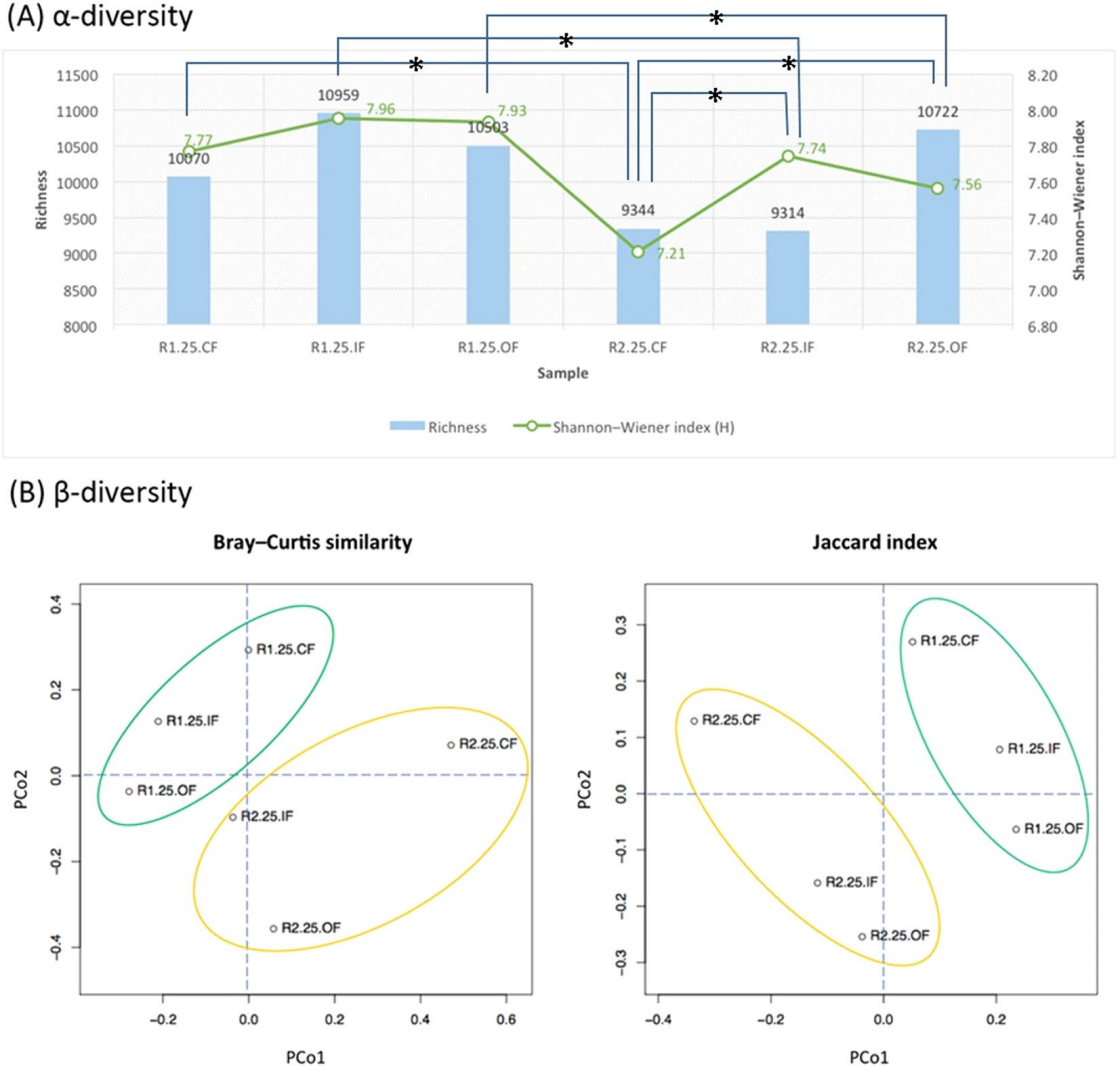
Community analysis of bacterial 16S OTUs in six soil types (R1.25.CF, R1.25.IF, R1.25.OF, R2.25.CF, R2.25.IF, and R2.25.OF). (**A**) The α- diversity was estimated by the number of observed species Sobs and Shannon–Wiener index (H’). The diversity was significantly higher in the R1 than R2 systems under same fertilization treatment. R2.25.OF and R2.25.IF was also substantially higher than R2.25.CF, respectively, based on Wilcoxon’s signed rank statistics (one-tailed), and: *P < 0.05 was found between different treatment. (**B**) Count-based distance metrics based on Bray–Curtis dissimilarity and Jaccard index for principal coordinate analysis (PCoA) were visualized in biplots. Soils treated with OF and CF were located on the opposite site of the second PCo axis, while the IF soils fall in between.

**Figure 3:**
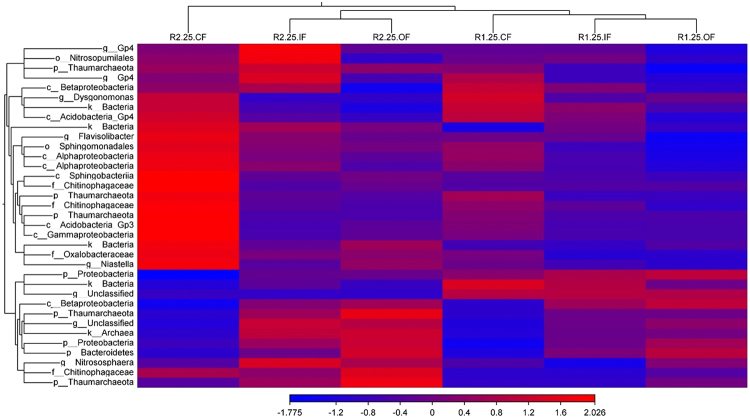
Heatmap with relative abundance based on read counts of top 35 16S OTUs grouped using the Euclidean distance average linkage criteria among six soil types (R1.25.CF, R1.25.IF, R1.25.OF, R2.25.CF, R2.25.IF, and R2.25.OF). The abundance OTUs mainly clusters depending on the rotation system and subgroups IF and OF among the two systems. Some of these OTUs (i.e. *Flavisolibacter* and *Sphingomonadales*) are highly expressed in R2.25.CF, but rarely in R1.25.OF, and vice versa (i.e. *Proteobacteria* and *Betaproteobacteria*).

Based on 17,683 high-quality OTUs, the bacterial α-diversity was taken into account on both species richness and evenness, and the diversity index was relatively higher in the R1 system, as shown in Fig. 2A. This may also be explained by the fact that highly accumulated mineral elements and nutrients (e.g. organic matter) (as displayed in Fig. S2) response to diverse microbes in R1 and OF. To compare the composition of microbial community in these six soil types, count-based distance metrics based on Bray–Curtis dissimilarity and Jaccard index for principal coordinate analysis (PCoA) were visualized in biplots^17^. As demonstrated in Fig. 2B, the successful increase in bacterial β-diversity came with different types of rotation system, which separates the composition of operational taxonomic units (OTUs) into R1 and R2 groups. Moreover, the distance analysis from OTUs certainly suggests that the microbial communities of IF is more similar to OF than CF, but quite distinct between OF and CF. Furthermore, the Venn diagram (Fig. S3) supports the notion that OF closer to IF than CF as well. We further analyze the top 35 OTUs based on the total counts among six soil types. The heatmap of these soils is also grouped by rotation systems, and OF is closer to IF in both systems (Fig. 3). The results were further verified by the PERMANOVA analysis, which showed that OF is closer to IF (with no significant difference (*P* = 0.07413)) (Table S3). In summary, our finding explicitly demonstrates that the rotation system determines the microbial composition, and the biodiversity of the field using integrity fertilizers is closer to that using organic than to chemical ones.

### Identification of enriched microbes by novel taxonomic enrichment analysis

The taxa existing in the soils are further unraveled, since the diversity and composition of the microbial communities are quite different among the six soil types and strongly driven by the rotation system. A total of 68, 56, and 54% 16S OTUs corresponding to CF, IF, and OF soils are assigned to bacteria at the phylum level (Fig. S4). Among them, the *Proteobacteria* (25%) and *Bacteroidetes* (19%) phyla are more abundant in CF than OF soils, whereas the most dominant phylum in OF soils is *Acidobacteria* (47%). When considering the taxonomic composition in the six soil types individually, the bacteria compositions among CF, IF, and OF are similar in the R1 system, except *Bacteroidetes* is much more common in CF. Notably, the majority of *Proteobacteria*, *Bacteroidetes*, and *Verrucomicrobia* phyla in CF soils mainly come from the R2 system. In addition, the OF soils in the R2 system have less *Acidobacteria* and more *Proteobacteria*, *Bacteroidetes*, and *Verrucomicrobia* phyla than in the R1 system. Surprisingly, the composition of dominant bacteria was similar in R1.25.CF and R2.25.OF, implying explicitly that paddy-upland rotation provides a best alternative to organic farming for sustainable agriculture if the use of chemical fertilizer is still required. Although many factors should be considered when assessing the best way for sustainable agriculture, paddy-upland rotation system can really recover partial negative effect from chemical fertilizer in the view of microbiome.

The taxonomy-assigned OTUs were estimated using their relative abundance at a fixed rank (e.g. phylum, class, and family) in most of the previous studies^10,16^. However, this strategy limits the determination of enriched microbes at the exact taxonomic ranks they belong to. Here, we developed a novel approach to identify taxa that were enriched in the OF and CF soils. The OF- and CF-enriched microbes with a cumulative hypergeometric *P*-value < 0.01 in the R1 and R2 systems are shown in Tables S4–S7. In the top 10 OF-enriched OTUs (compared with CF), one of the *Acidobacteria*, subgroup *Gp6* identified at the genus level, is dominant in both R1OF and R2OF. The *Actinobacteria* is also the dominant phylum in R1OF and R2OF, and most of its genus *Streptomyces* may have a function in disease resistance^18^. In contrast, three phylum of bacteria, *Verrucomicrobia*, *Proteobacteria* and *Bacteroidetes*, were dominant in R1CF and R2CF soil, as shown in Tables S4–S7 and Fig. S4, respectively.

### Identification of OF-, CF-, and R1-specific bacteria

**Figure 4:**
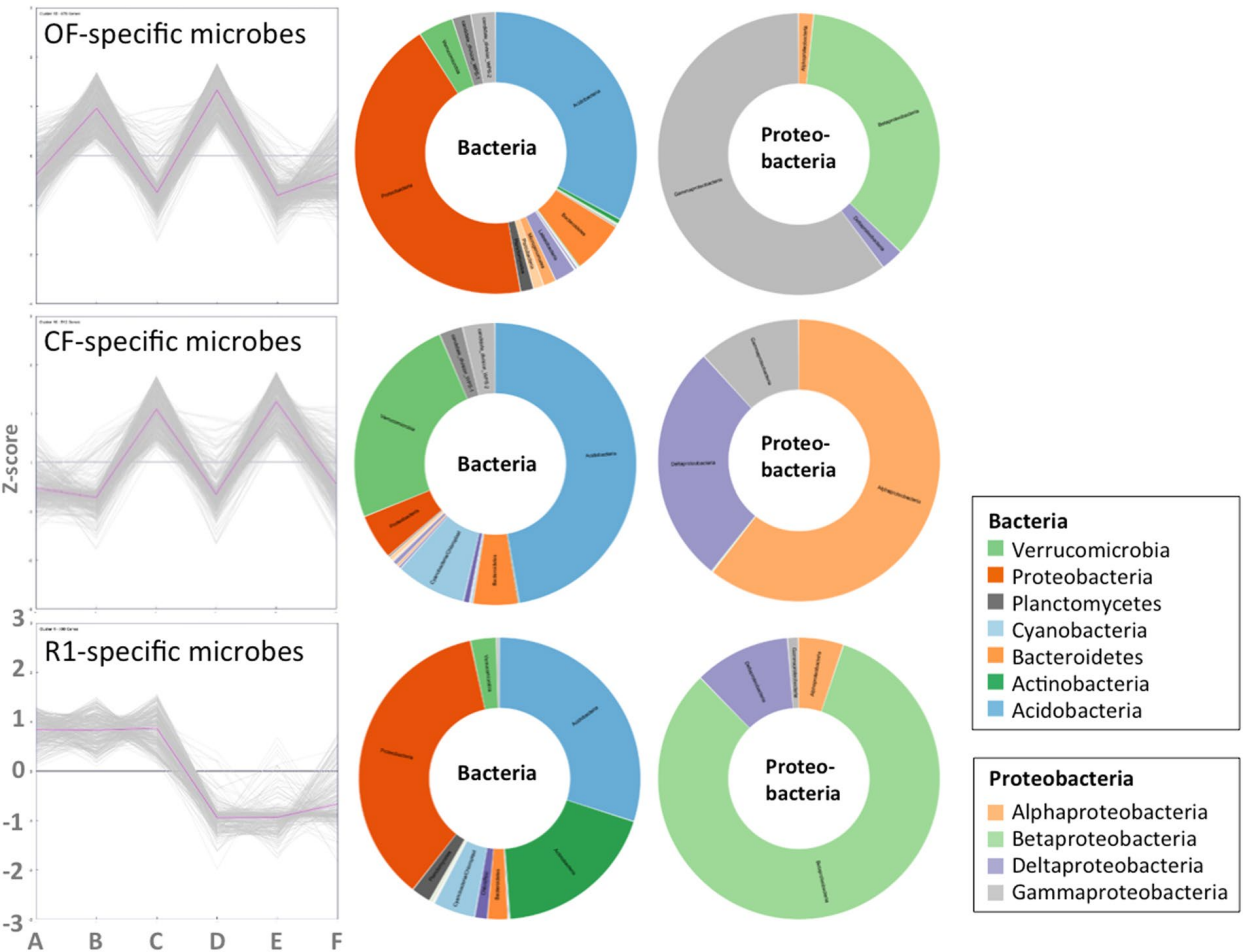
The composition of OF, CF, and R1-specific co-occurrence microbes based on k-means clustering for taxonomy-assigned 16S OTUs in six soil types (A: R1.25.IF, B: R1.25.OF, C: R1.25.CF, D: R2.25.OF, E: R2.25.CF, and F: R2.25.IF). The normalized read counts of OTUs among these were transformed to z-scores. Eventually, 679, 512, and 408 co-occurring OTUs with similar patterns were identified in the OF-, CF-, and R1-specific groups, respectively. The co-occurrence patterns of OF, CF, and R1-specific microbes are shown in the left three plots (pink lines represent the average z-score). The composition of the bacteria and *Proteobacteria* is shown in sunburst charts.

A new application of co-occurrence method was also developed and utilized in this study to amplify the pattern of OTUs expression with the same rotation or fertilization groups. K-means clustering was applied to classify the taxonomy-assigned 16S OTUs with similar appearance patterns into co-occurrence groups as specific microbes. Eventually, 679, 512, and 408 co-occurrence OTUs with similar patterns are identified in the OF-specific, CF-specific, and R1-specific groups, respectively (Fig. 4, left). Interestingly, the composition of OF-specific bacteria is relatively abundant in *Proteobacteria* and *Acidobacteria*, similar to the R1-specific group, whereas the CF-specific bacteria had a very different pattern of relative abundance (less *Proteobacteria* but more *Verrucomicrobia*). More abundant *Proteobacteria* phylum was found in the OF- and R1-specific groups, at the class level, *Betaproteobacteria* were the most common microbes. In contrast, *Alphaproteobacteria* and *Deltaproteobacteria* were predominant in the CF-specific group, but none *Betaproteobacteria* has been identified.

### Correlation with disease severity and microbial diversity

**Figure 5:**
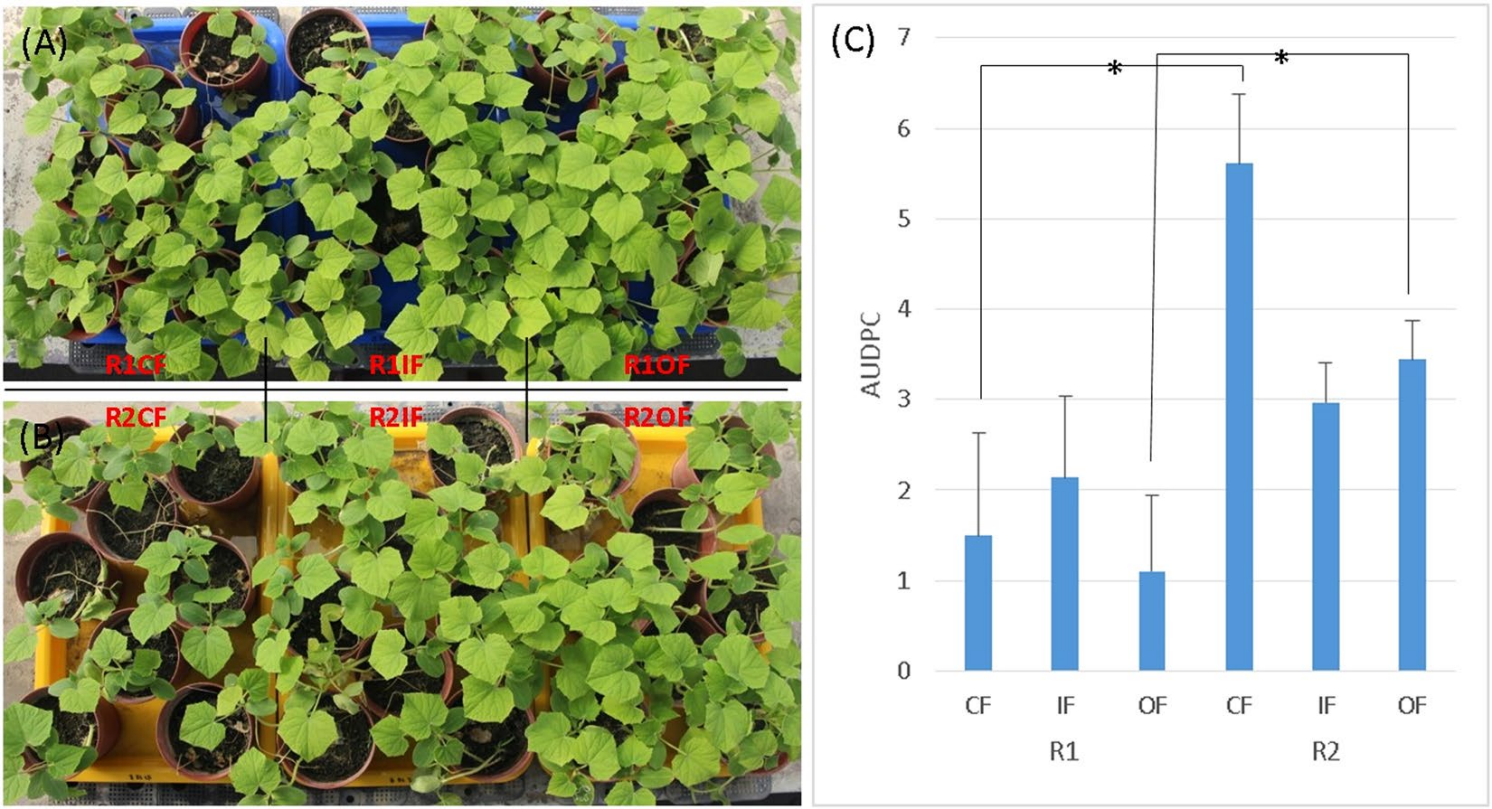
Assessing the disease incidence of six soil types. (**A**,**B**) R1 and R2 soils, respectively, with fertilization in the order of CF, IF and OF from left to right, (**C**) the AUDPC assesses among the six soils. An asterisk above the bars indicates a statistically significant difference (*P* < 0.05, Wilcoxon’s signed rank statistics), and *P < 0.05, **P < 0.01, ***P < 0.001 was found between two soils.

The damping off disease was respectively assessed by cucumber seedling in a series pot bioassay and cabbage in experiment field, to evaluate the healthy status of these soils. The disease progress of *Rhizoctonia* damping-off on cucumber seedlings in R1OF soil had the lowest incidence (Fig. S5). Adding one plug of mycelium pathogen, a notable difference also discovered in comparison with R2OF and R2CF (*P* < 0.01) (Supplementary Information Fig. 4). Translating the disease incidence into AUDPC, we found the AUDPC of CF and OF between R1 and R2 under natural conditions were significantly different (*P* < 0.05), and less disease incidence were found in R1 than R2 system (Fig. 5). In addition, a notable difference was demonstrated within the R2OF and R2CF (P < 0.01) (Fig. S6). The results suggest that the lowest and highest AUDPC has been identified in R1OFand R2CF, respectively. Further analysis of the correlation between AUDPC and microbial diversity showed a remarkably negative correlation via the polynomial regression (Fig. S7A), suggesting greater soil diversity leads to less disease incidence. In addition, the field survey illustrated that after counting 17,312 cabbage seedlings, the incidence of damping off disease in each field was also significantly and inversely correlated with microbial diversity (Fig. S7B). Expectedly, when one plug of mycelium pathogen was added into R1OF soil, the value of AUDPC did not increase. It is accordingly demonstrated that the R1OF soil had the capability to resist biological disturbance and stress.

### Assessment of disease-suppressive microbes

The correlation between the assigned 16S OTUs and AUDPC among the soils was further analyzed. Surprisingly, based on the combination of total read counts, the top 20 OTUs (r <= −0.8) affiliated to *Acidobacteria*, *Proteobactria* and *Actinobacteria* were still the top three most abundant phyla in R1 and OF soils (Table S8). Three representative phyla had the highest common factor with regard to the previous relative abundance data (Fig. S4), enrichment analysis in R1OF and R2OF (Tables S4–S7), and co-occurrence for the R1 and OF soils (Fig. 4). Within these phyla, some specific microbes were identified at the order or genus taxonomic levels, such as the *Proteobactria* subgroup *Burkholderiales* following *Acidobacteria* subgroup *GP16*, *Actinobacteria* subgroup *Streptomyces*, and *Acidobacteria* subgroup *GP6*. Moreover, the correlation between the top 20 OTUs and AUDPC (r > = 0.8) (e.g. p__*Thaumarchaeota*, o__*Sphingomonadales*, and f__*Chitinophagaceae* etc.) in Table S9 was very similar to the relative abundance microbes in R2CF (Fig. 3).

## Discussion

In this study, we find the microbial diversity and composition is highly determined by rotation manipulation rather than fertilizations. The soil properties of R1 and R2 show that organic fertilization significantly improve accumulation of organic matter, and increase chemical fertility in the long-term experiment (Fig. S2), consistence with previous studies^19–21^.

In addition, the higher organic matter and chemical element indeed harbor higher diversity of microbes (Fig. 2), especially in paddy-upland rotation system. Reciprocal cultivation between flooding and drying under anaerobic and aerobic conditions could change the soil C and N cycles and soil physical properties, presumably explaining the increase in microbial diversity with a paddy-upland rotation system. These results firstly suggest that regardless what fertilizers employed, the bacterial diversity in soils would be higher with the use of paddy-upland rotation rather than general upland rotation, while organic farming brings more biodiversity than chemical fertilizers in both rotation systems. It is worthy to note that the bacterial diversity is higher in both OF and IF than that in CF in the same rotation system (Fig. 2A). This indicates implicitly that the less microbial composition of chemical fertilizers (CF) is not suitable for sustainable agriculture. As a result, if the fully organic fertilization cannot be applied, integrated fertilization (i.e. half organic/inorganic) could be considered.

The bioinformatics approaches applied in this study overcome the restriction of the enriched taxa analysis on metagenomics sequencing data. The taxa of all levels are considered. Numerous OTUs of *Bacteroidetes* come from CF soil (Fig. S4A) are also consistent with observation in the relative abundance in nitrogen fertilization soil^22^. *Bacteroidetes* are regarded to degrade high molecular weight organic matter^23^, the role of this bacteria in CF soil still needed further study. *Betaproteobacteria*, one of the *Proteobacteria* subgroup is a notable class for efficient nitrogen fixation^24^, and among them, the genus *Burkholderia* spp., promotes highly plant growth^18^. *Actinobacteria* is also the dominated phylum in R1OF (Table S4) and R1-specific (Fig. 4). *Actinobacteria* is well-known for its decomposition of organic matter in agriculture soils, and most of the genus *Streptomyces* makes its contribution for promoting plant growth and secretion of antibiotics against the plant pathogens in soil^18^. Besides, *Planctomycetes* only appear abundance in R1OF, which have a unique anaerobic, autotrophic metabolism to oxidize ammonium^25^, associated with aerobic degradation of plant saccharides, and their ecological role are commonly associated with algae^26^, consistence with the paddy-upland belonging to wetland environment in this study.

Comparing with the rotation systems, R1 soil have higher microbial diversity than R2 does (Fig. 2). R1 soils have lower disease incidence than R2 soils, whenever conduct pot bioassay (Figs 5, S5, S6) or field survey (Fig. S7). Although there are no significant differences in disease incidences between two rotation systems in IF treatment, the microbial diversity and disease incidence of six treatments have proved with high correlation (R^2^ = 0.8816) as taking two IF treatments (R1IF and R2IF in consideration (Fig. S7A). This finding are consistent with Grube *et al*., who suggested that maintaining microbial diversity in the different environments is an important issue to avoid pathogen outbreaks for plant and human^27^. The *Burkholderiales* and the *Streptomyces* identified in R1 and OF are both best for their benefits in agriculture soil, promoting plant growth and antifungal activity^28^. Interestingly, the *Actinobacteria* is higher in R1-specific than OF-specific and R2-specific bacteria, respectively (Figs 4 and S4), referring the advantages of R1 rotation system. By global screening of the antagonistic activity towards *Rhizoctonia solani* from the suppressive soil, a large proportion of *Streptomyces* spp. was identified^29^. These bacteria (e.g. *Streptomyces*) were deduced as the key microbes to suppress the *Rhizoctonia* damping off disease of cucumbers, and had been used in the control of *Rhizoctonia solani* in tomatoes^30^. Moreover, *Acidobacteria* subgroups *GP16* and *GP6* identified in this study play essential roles against pathogens. Several specific microbes such as *Burkholderiales* and *Streptomyces* could be isolated from the soil, and the bacteria compositions of R1OF provides an indicator for microbial agents. Taken together from soil properties, biodiversity and disease management, paddy-upland rotation manipulation is highly recommended for sustainable agriculture.

## Methods

### Experiment field and fertilization

The experiment field is located at the Kaohsiung District Agricultural Improvement Station in Kaohsiung County (22°51′28.1″N 120°30′58.4″E), Taiwan. The climate is that of a tropical region (annual average temperature: 20.4~29.6 °C, annual precipitation: 2,200 mm). The original field soil was hyperthermic, udic, haplaquept, mixed and calcareous, with a silty loam texture. The original main plot field was designed for two kinds of crop rotation systems: the paddy-upland rotation of rice−rice−vegetables was implemented in Rotation 1 (R1), whereas Rotation 2 (R2) chose the consecutive upland utilization of sweet corn−sesbania−vegetables during the growing season (Fig. 1). Three fertilization treatments were employed for each main rotation system, including organic (OF), chemical (CF), and a combined (1:1 organic to chemical) fertilizers (IF), based on same amount of N. The plot size of each fertilization treatment was 0.1 hectare. The amounts of chemical fertilizers and composted manure (organic fertilizer) applied for crops were described in a previous paper^31^. In brief, the corresponding treatments for the present study were the same as in every previous year. The application of chemical fertilizer was carried out in the CF plot with the use of urea for N, superphosphate for P and potassium chloride for K. The total amount of N applied in the OF plot was twice that of the CF plot. The actual amounts of organic fertilizer applied on vegetables varied (~20,000 kg/ha) depending on the water content within the vegetables. Each corresponding treatment was kept constant for every cropping season.

### Soil sampling and chemical analysis

Each plot was divided into four subplots for sampling. A total of 24 soil samples in six long-term farming soils (R1.25.CF, R1.25.IF, R1.25.OF, R2.25.CF, R2.25.IF, and R2.25.OF) were collected from the superficial layer (0–15 cm) in January 20^th^, 2014, after the same vegetable crop variety had been harvested. All soils were kept on dry ice and then stored at −20 °C until use. The chemical properties of soil were analyzed using a method described previously^32,33^. Assessment of any significant difference in the essential elements among the six soil types was performed using the SAS-EG software package (v7.1) for statistical analyses.

### DNA extraction, library preparation, and sequencing

The soil DNA from each sample was extracted using a PowerSoil® DNA Isolation Kit (MO BIO) after adding 0.25 grams of soil sample as the starting material according to the manufacturer’s instructions. DNA purity and concentration (ng/μl) were determined by the ratio of the absorbance at 260 and 280 nm (A260/280), using Nanodrop and a Qubit® 3.0 Fluorometer (Thermo Fisher Scientific Inc.) to ensure the extracted soil DNA was suitable for the following steps. Next, the polymerase chain reaction (PCR) amplicons for bacterial 16S_V4−V5_ hypervariable region of ribosomal RNA genes were successfully generated using a Phusion® High-Fidelity PCR Master Mix with HF Buffer (New England Biolabs) and specific primer sets F515/R806 acquired from previous studies, respectively^34^. The PCR conditions are explicitly described in Table S1. Before sequencing, amplicon DNA was purified, end repaired and A-tailed using the polymerase activity of known fragments, ligated using indexed adapters, and validated against the libraries using a QPCR, Qubit, and Experion™ automated electrophoresis system (Bio-Rad). Finally, 2 × 250 bp paired-end sequencing for each amplicon library was conducted on the Illumina Miseq platform. Sequence files were submitted to the NCBI Sequence Read Archive (www.ncbi.nlm.nih.gov/sra) with BioSample accession number SAMN 07728445.

### Metagenomic analysis pipeline

A metagenomic analysis pipeline was constructed to distinguish microbiomes and their community structures surveyed (Fig. S1). The preprocessing of raw sequencing reads derived from 16S rRNA libraries was performed on a CLC Genomics Workbench 8.5 and 9.0 (QIAGEN). Briefly, quality trimming based on the quality score Q30 was accomplished after removing adaptors. The overlapping forward and reverse pairs with identical barcodes were then merged and demultiplexed to prevent unexpected chimera for full-length sequences within their corresponding soil samples. Afterwards, 32-bit USEARCH (v8.1.1861) and mothur (v.1.37.0) were used to execute the steps of fixed length trimming, dereplication, discarding singletons, chimera filtering, *de novo* OTU clustering (97% similarity), and taxonomy assignment^35,36^. The reference databases for 16S classification, RDP trainset 15 and UNITE v7, were downloaded directly from the UPARSE official website (drive5)^37^. All the parameters were set by default or at the suggested values. In total, 3,123,368 demultiplexed full-length reads merged from paired-end sequencing of bacterial 16S_V4~V5_ rRNA libraries was obtained after the quality trimming procedure. All the high-quality bacterial sequences were clustered into 17,683 operational taxonomic units (OTUs) at the 97% similarity level by excluding singletons and possible chimera.

### Community analysis

#### Statistics

Prior to the statistical analysis for the microbial community, the OTU table was normalized by dividing the read counts of detectable OTUs over the total number of sequences for each sample. The diversity of the bacterial communities within (α-diversity) and between (β-diversity) soil samples was estimated by the functions of *vegan*, *ape*, and *ecodist* packages in R. Besides, the number of observed species *S*_*obs*_, using the Shannon–Wiener index (H’), was calculated to reflect the evenness of species within a sample. Count-based distance metrics for principal coordinate analysis (PCoA) were established based on Bray–Curtis dissimilarity and Jaccard index. In addition, the relative abundance of dominant OTUs was visualized using sunburst charts, heatmap and PERMANOVA analysis via CLC Microbial Genomics Module with an evaluation license. Assessment of the Shannon–Wiener index (H’) diversity and area under disease progress curve (AUDPC) were performed with nonparametric analysis by Wilcoxon signed-rank test^15^ using the SAS-EG software package (v7.1) for the statistical analyses. The relationships between AUDPC and microbial diversity were evaluated by polynomial regression analyses.

#### Taxonomic enrichment

Based on the taxonomy annotations of assigned 16S OTUs sourced from UPARSE, the analysis of taxonomic enrichment was performed to determine the OF- or CF-enriched microbes (the fold change of OF/CF >2 and <0.5, respectively) in R1 and R2 rotation systems, respectively. Since each taxon occupies a strict taxonomic rank in the hierarchy, the cumulative *P*-value of the hypergeometric distribution was calculated using the following formula:

1 $${p}({X}\le {k})={\sum }_{{i}={x}}^{{n}}\frac{(\frac{{M}}{{i}})(\frac{{N}-{M}}{{n}-{i}})}{(\frac{{N}}{{n}})}$$

where ***N*** and ***M*** denote the number of OTUs with taxonomy assignment and the total OTUs assigned to the specific taxonomy, whereas there are ***i*** OTUs out of ***n*** OTUs with that taxonomy in the group ***X***.

#### Identification of co-occurrence microbes

In this study, co-occurring microbes were defined as a group of microorganisms that appear simultaneously as well as have a similar existence trend in various sampling sites. For identifying co-occurring microbes in the OTU table, the normalized reads of OTUs among six soils were transformed to z-scores. OTUs with similar appearance patterns were then grouped together by non-hierarchical algorithms using the k-means clustering method^38^, with high-throughput gene expression analysis applied to classify taxonomy-assigned 16S OTUs with similar appearance patterns into co-occurrence groups. The composition of co- occurring CF, OF, and R1-specific microbes was further surveyed.

#### Assessment of the disease incidence of soils

The six soil types for pot bioassay were first collected on January 20^th^, 2014. The results were doubly confirmed using soils collected on Jun. 25^th^, 2016. The fresh soils from the second collection date were tested for cucumber seeds (*Cucumis sativus* L. c.v. Kappa 11) bred in flowing water for 4 h, then placed on and finally covered with wetted-tissue paper overnight before sowing. After root extension, the seedlings were sown in PVC pots (diameter 8 cm; height 7 cm; 10 seedlings/plot) containing 300~400 g of soil with an initial moisture content above 80% (v/w). Plants were grown in a plastic green house at room temperature in June (nearly 27~35 °C, 70% relative humidity), and watered daily with the same amount of water in each pot (nearly 50~100 ml/pot/day depended on the weather conditions). The trial was set for four treatments: i) six soil types (R1/OF,IF,CF; R2/OF,IF,CF) alone; ii) soil amended with fungal pathogen (*Rhizoctonia solani*) (purchased from The Food Industry Research and Development Institute (FIRDI), Taiwan) by transferring one to two mycelia agar plugs (5-mm-diameter) from a 1 week-old potato dextrose agar (PDA) culture at 1-cm underneath the soil surface according to previous report^39^; iii) soil sterilized at 121 °C for 30 min as a blank control; and iv) sterilized soils amended with fungal pathogen by transferring one plug of mycelia agar. For each soil treatment, eight pots (replicates) were set in a completely randomized experimental place. The number of infected cucumber seedlings was scored as disease incidence every 2–5 days for a period up to 14 days after pathogen inoculation. The translation of disease incidence to AUDPC was determined according to statistical methods^40^. The correlations between AUDPC and the Shannon–Wiener index (H’), and the OTUs among the six soil types, were obtained using polynomial regression. To confirm the correlation between microbial diversity and disease incidence, 21-day cabbage seedlings (*Brassica oleracea* L. var. Capitata, L. c.v. chu- chiou) purchased from a commercial nursery were planted in autumn cropping season. A survey was conducted by counting the number of cabbage seedlings damping off in each field on the seventh day after planting.

### Data Availability

The datasets used and/or analyzed during the current study are available from the corresponding author on reasonable request.

## Electronic supplementary material

SUPPLEMENTARY INFORMATION
