# Supplementary material for: Paddy-upland rotation for sustainable agriculture with regards to diverse soil microbial community
Source: Sci Rep. 2018 May 22;8:7966. doi: 10.1038/s41598-018-26181-2 (PMC5964091; doi:10.1038/s41598-018-26181-2)
Supplement: Supplementary file 1 — SUPPLEMENTARY INFORMATION [file 41598_2018_26181_MOESM1_ESM.docx]

**SUPPLEMENTARY INFORMATION**

**Paddy-upland rotation for sustainable agriculture with regards to diverse soil microbial community**

Ping-Fu Hou, Chia-Hung Chien, Yi-Fan Chiang-Hsieh, Kuan-Chieh Tseng, Chi-Nga Chow, Hao-Jen Huang*, and Wen-Chi Chang*

*correspondence to: sarah321@mail.ncku.edu.tw


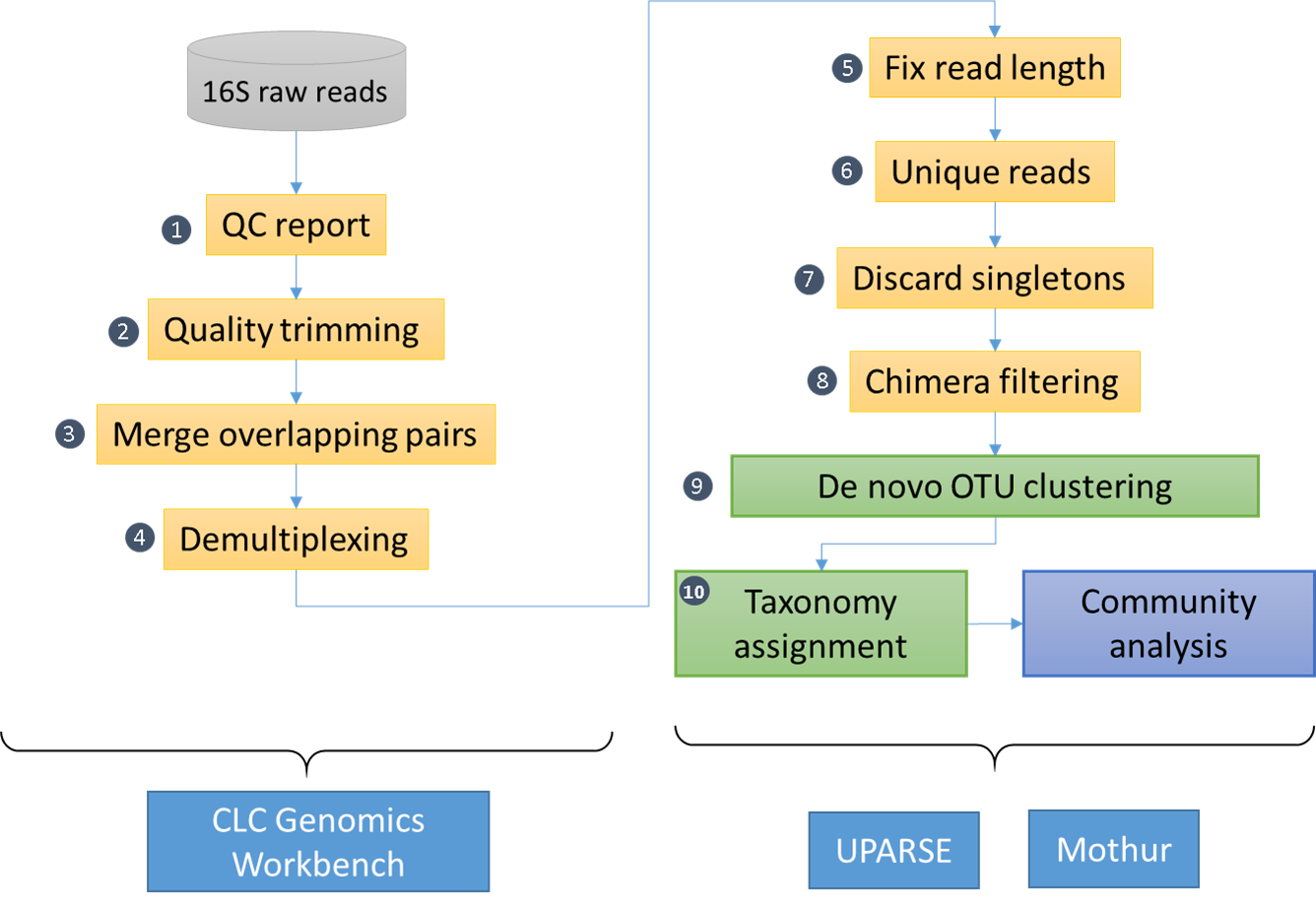


Figure S1 The metagenomic analysis pipeline. The preprocessing of raw sequencing reads derived from 16S rRNA libraries was performed on CLC Genomics Workbench 8.5 and 9.0 (QIAGEN), and the rest of the steps were conducted using 32-bit USEARCH (UPARSE)(v8.1.1861) and Mothur (v.1.37.0).


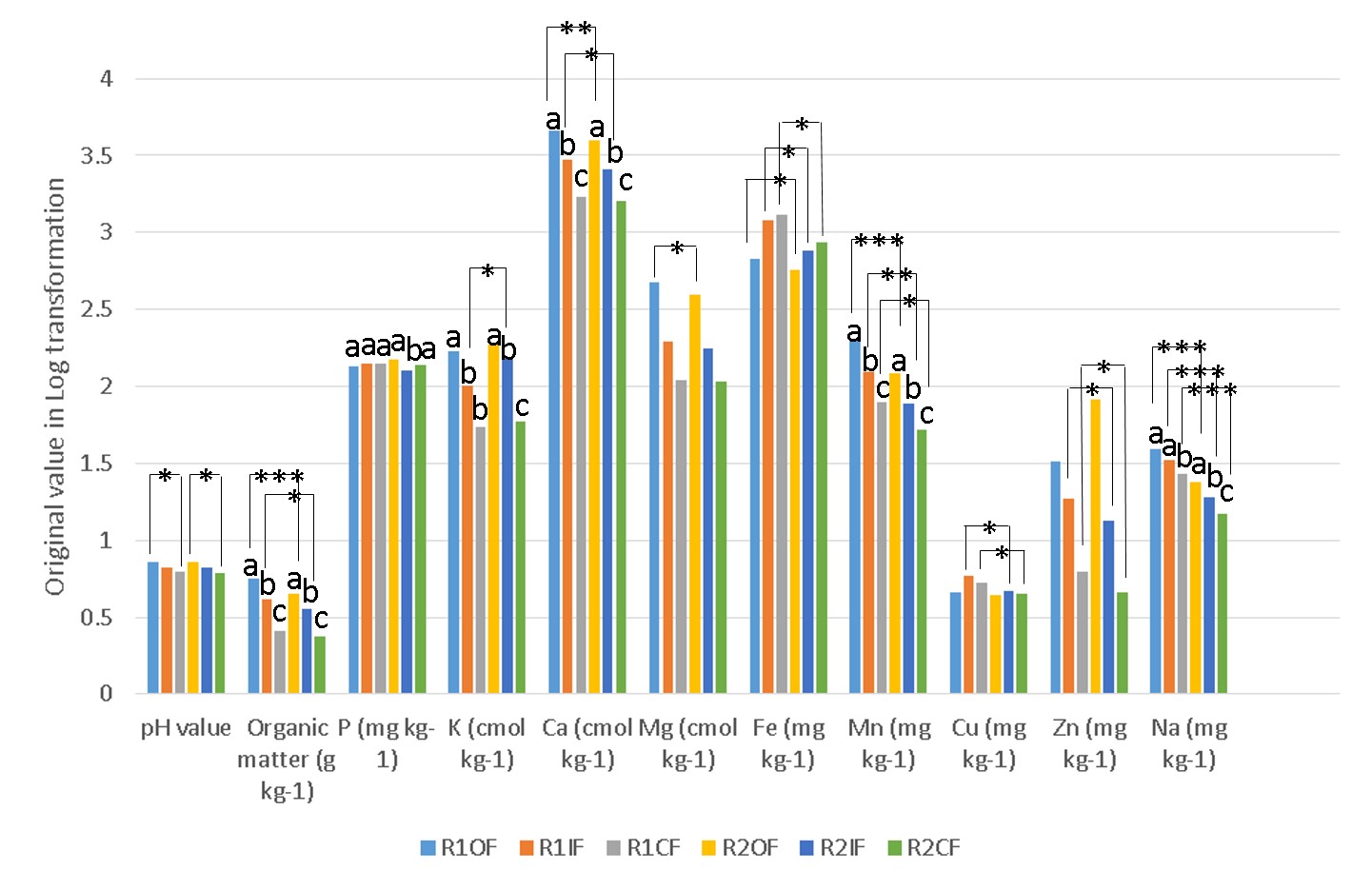


**Figure S2** Log transformation of the original mean properties of six sampled soil types. The chemical elements of the representative soils examined in this work show that organic matter, K, Ca and Mg accumulate significantly more in OF than IF and CF, and there are also significant differences between two rotation systems under the same fertilization regime. The values of organic matter, P, K, Ca, Mn and Na were examined by ANOVA analysis for the normal distribution, whereas pH, Mg, Cu and Zn were analyzed with nonparametric analysis by Wilcoxon’s signed rank statistics (one-tailed). Different letters above the bars indicate statistically significant differences (*P*<0.05, LSD test), and *:*P*<0.05, **:*P*<0.01, ***:*P*<0.001 was found between R1 and R2 under same fertilization treatment or between OF and CF (i.e. pH value).


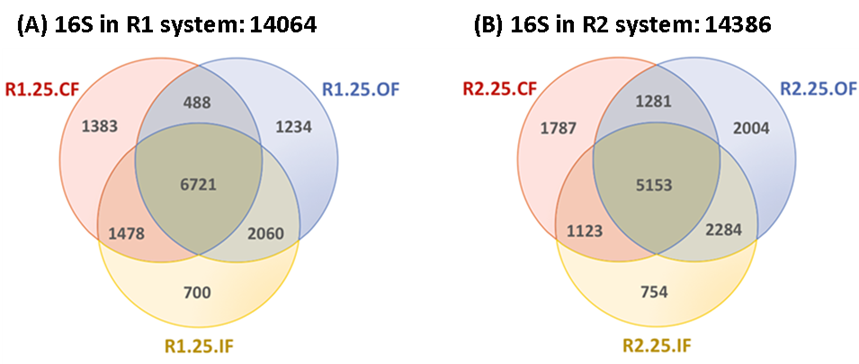


Figure S3 The Venn diagram with three sets of 16S OTUs among OF, IF, and CF soils in the R1 (A) or R2 (B) system. The number of 16S OTUs in the intersection of OF and CF was much lower than that in the OF-IF intersection in both rotation systems (488 vs. 2060 and 1281 vs. 2284). OF soils apparently shared more 16S OTUs with CF soils in the R2 system (1281 vs. 488).


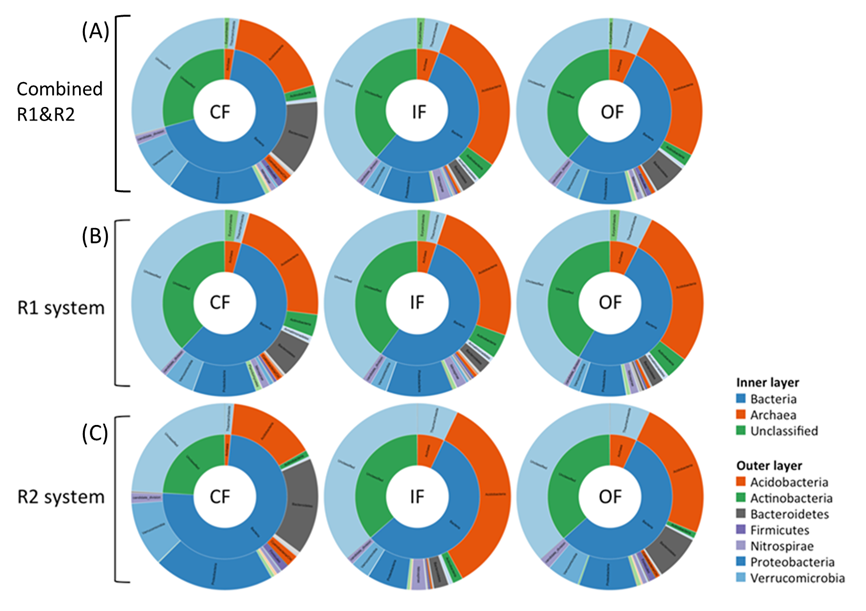


Figure S4 Relative abundance of all assigned 16S OTUs. (A) the upper three sunburst charts demonstrate the relative abundance of 16S OTUs at the phylum level in CF, IF, and OF soils (combined R1 and R2 systems), whereas the middle (B) and bottom (C) sunburst charts show the relative abundance of 16S OTUs in the R1 and R2 systems separately. The inner layers of the sunburst charts indicate the proportion of bacteria, archaea, and unclassified OTUs. The outer layers display the composition and relative abundance of the dominant microbes belonging to bacteria, archaea, and unclassified OTUs. Among them, the *Proteobacteria* (25%) and *Bacteroidetes* (19%) phyla are more abundant in CF than OF soils, whereas the most dominant phylum in OF soils is *Acidobacteria* (47%).


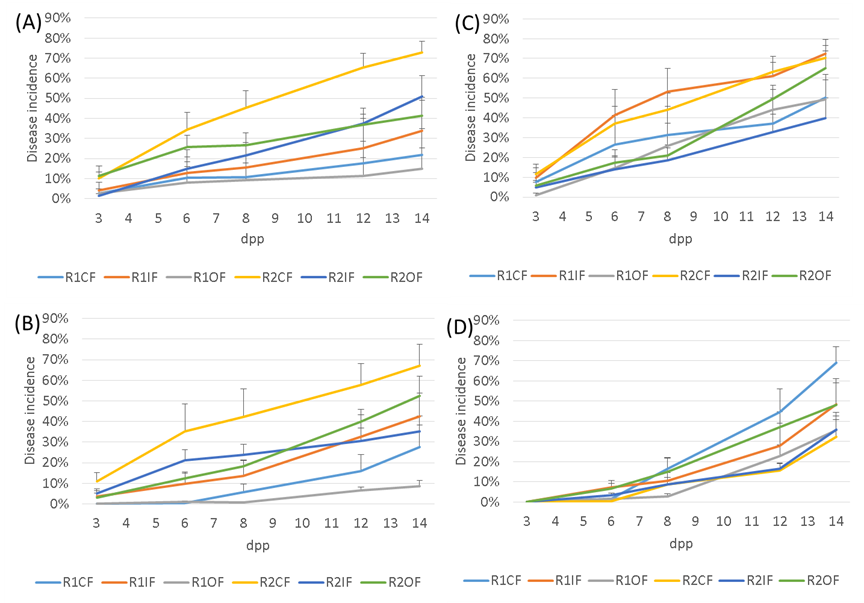


Figure S5 Progress of *Rhizoctonia* damping off disease of cucumber seedlings among six type soils. Disease incidence (%) was calculated every 2-5 days post plating (dpp) in pot bioassay (mean values ±SEM, N = 8). (A) Soils without sterilization and inoculation. (B) Soils without sterilization and inoculation with 1 disc mycelium plug of *Rhizoctonia solani*. (C) Soils without sterilization and inoculation with 2 disc mycelium plug of *Rhizoctonia solani*. (D) Soils with sterilization and inoculation with 1 disc mycelium plug of *Rhizoctonia solani*.


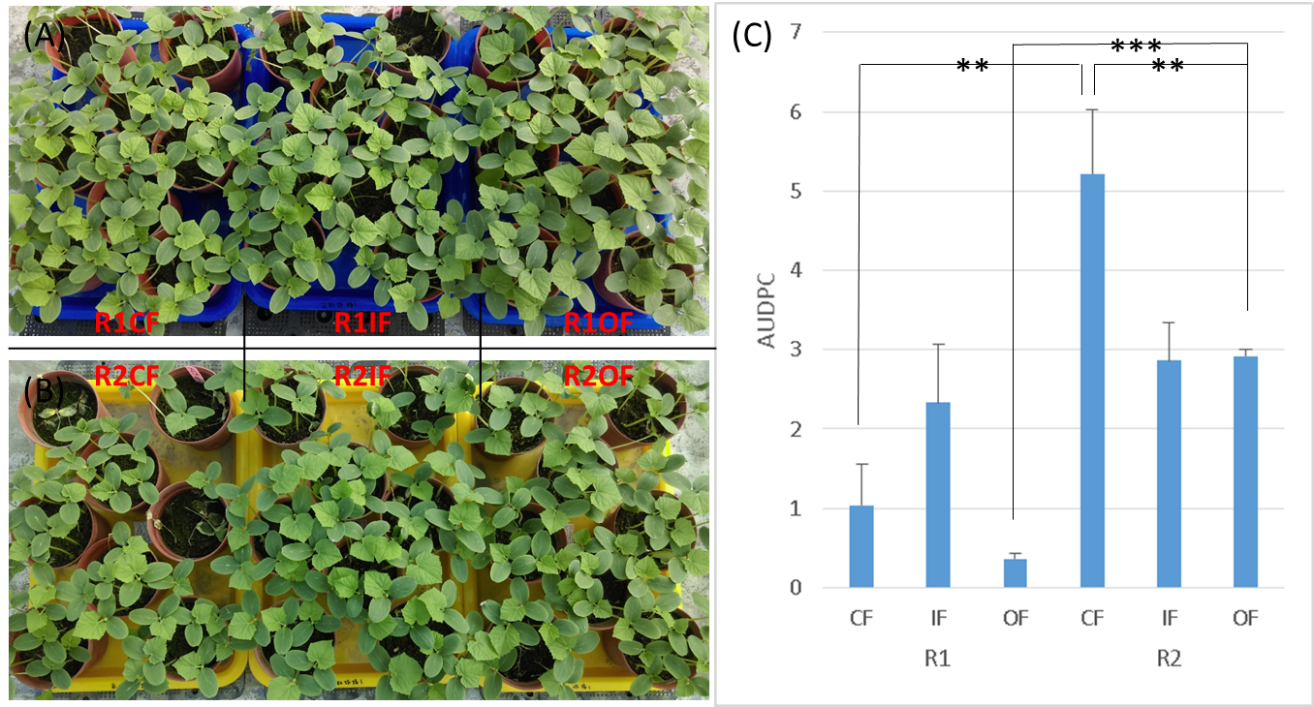


Figure S6 Comparison of AUDPC among six soil types amended with one disc plug of mycelium *Rhizoctonia solani*. AUDPC were calculated in the term of observation in pot bioassay (mean values ±SEM, N = 8). An asterisk above the bars indicates statistically significant differences (*P*<0.05, Wilcoxon’s signed rank statistics), and *:*P*<0.05, **:*P*<0.01, ***:*P*<0.001 was found between two soils.

(A) (B)

| 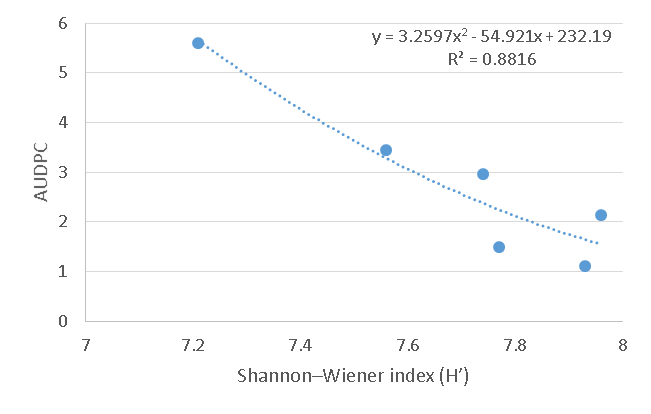 | 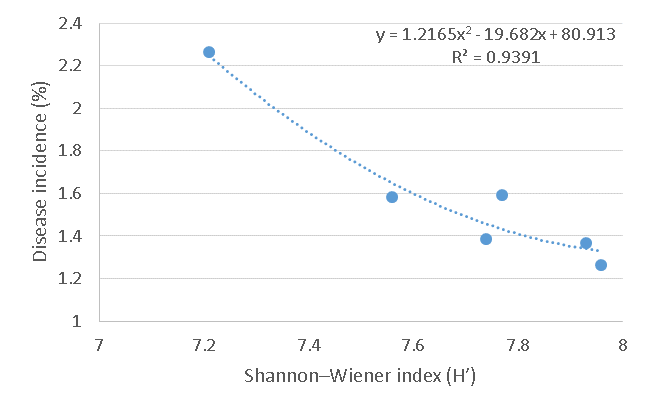 |
| --- | --- |

Figure S7 The correlation of AUDPC and Shannon-Wiener index (H’) in polynomial regression. AUDPC and disease incidence data come from the pot bioassay of cucumber (A) and field survey of cabbage (B), respectively.

Table S1 The PCR conditions used to amplify the hypervariable regions of 16S rRNA genes.

| Amplicon | PCR condition |
| --- | --- |
| Bacterial 16S_V4-V5_ | 94°C, 3 min; |
|  | 35 cycles at 94°C for 45 s, 50°C for 60 s, and 72°C for 90 s; |
|  | 72°C, 10 min |

Table S2 The properties of six sampled soil types.

| Soil types | pH value | Organic matter (g kg^-1^) | P (mg kg^-1^) | K (cmol kg^-1^) | Ca (cmol kg^-1^) | Mg (cmol kg^-1^) | Fe (mg kg-1) | Mn (mg kg-1) | Cu (mg kg^-1^) | Zn (mg kg^-1^) | Na (mg kg-1) |
| --- | --- | --- | --- | --- | --- | --- | --- | --- | --- | --- | --- |
| R1.25.OF | 7.23 | 5.59 | 134 | 170 | 4537 | 470 | 667.0 | 204.5 | 4.57 | 32.7 | 39.5 |
| R1.25.IF | 6.69 | 4.12 | 141 | 101 | 2976 | 197 | 1204 | 123.5 | 5.92 | 18.5 | 33.5 |
| R1.25.CF | 6.27 | 2.58 | 141 | 54.5 | 1717 | 110 | 1311 | 78.75 | 5.35 | 6.22 | 26.7 |
| R2.25.OF | 7.29 | 4.52 | 150 | 187 | 3983 | 391 | 567.7 | 122.7 | 4.37 | 82.7 | 24.0 |
| R2.25.IF | 6.68 | 3.59 | 128 | 151 | 2546 | 178 | 767.5 | 77.25 | 4.72 | 13.5 | 19.0 |
| R2.25.CF | 6.16 | 2.36 | 138 | 58.7 | 1607 | 106 | 862.7 | 52.25 | 4.45 | 4.60 | 15.0 |

Table S3 PERMANOVA analysis by three distance measures between groups.

| Groups |  | *P*-value* |  |
| --- | --- | --- | --- |
|  | Bray-Curtis | Jaccard | Euclidean |
| R1, R2 | 0.00001 | 0.00001 | 0.00005 |
| OF, IF | 0.02984 | 0.01725 | **0.07413** |
| OF, CF | 0.00093 | 0.00093 | 0.00093 |
| IF, CF | 0.00093 | 0.00093 | 0.00047 |

* The null hypothesis depends on the Bonferroni method.

Table S4 Enriched microbes with the fold change of OF/CF > 2 in the R1 system. Only the microbes with hypergeometric *P*-value < 0.01 are shown. (All assigned taxa: 13256 / 17683, FC > 2: 2139)

| Taxa (taxonomy) | OTUs with taxa | OF-enriched OTUs with taxa | *P*-value |
| --- | --- | --- | --- |
| d:Bacteria;p:Actinobacteria | 534 | 159 | 5.80E-16 |
| d:Bacteria;p:Acidobacteria;c:Acidobacteria_Gp6 | 207 | 76 | 3.89E-13 |
| d:Bacteria;p:Acidobacteria;c:Acidobacteria_Gp6;g:Gp6 | 134 | 56 | 1.14E-12 |
| d:Bacteria;p:Acidobacteria | 1145 | 258 | 2.15E-09 |
| d:Bacteria;p:Acidobacteria;c:Acidobacteria_Gp17;g:Gp17 | 16 | 12 | 2.91E-07 |
| d:Bacteria;p:Actinobacteria;c:Actinobacteria | 347 | 88 | 5.46E-06 |
| d:Bacteria;p:Proteobacteria | 2470 | 472 | 6.81E-06 |
| d:Bacteria;p:Acidobacteria;c:Acidobacteria_Gp17 | 42 | 18 | 3.61E-05 |
| d:Bacteria;p:Proteobacteria;c:Betaproteobacteria | 318 | 78 | 6.29E-05 |
| d:Bacteria;p:Planctomycetes | 516 | 116 | 7.87E-05 |
| d:Bacteria;p:Ignavibacteriae | 34 | 15 | 0.000108 |
| d:Bacteria;p:Ignavibacteriae;c:Ignavibacteria | 28 | 13 | 0.000165 |
| d:Bacteria;p:Actinobacteria;c:Actinobacteria;o:Gaiellales | 17 | 9 | 0.000513 |
| d:Bacteria;p:Chloroflexi | 240 | 58 | 0.000772 |
| d:Bacteria;p:Proteobacteria;c:Alphaproteobacteria;o:Rhizobiales | 116 | 31 | 0.002441 |
| d:Bacteria;p:Actinobacteria;c:Actinobacteria;o:Gaiellales;f:Gaiellaceae | 11 | 6 | 0.00388 |
| d:Bacteria;p:Actinobacteria;c:Actinobacteria;o:Solirubrobacterales | 8 | 5 | 0.003968 |
| d:Bacteria;p:Acidobacteria;c:Acidobacteria_Gp7 | 50 | 16 | 0.00408 |
| d:Bacteria;p:Acidobacteria;c:Acidobacteria_Gp9 | 3 | 3 | 0.004196 |
| d:Bacteria;p:Chloroflexi;c:Caldilineae | 46 | 15 | 0.004381 |
| d:Archaea;p:Thaumarchaeota;o:Nitrososphaerales;f:Nitrososphaeraceae | 22 | 9 | 0.004882 |
| d:Bacteria;p:Ignavibacteriae;c:Ignavibacteria;o:Ignavibacteriales;f:Ignavibacteriaceae | 12 | 6 | 0.006707 |
| d:Archaea;p:Thaumarchaeota | 57 | 17 | 0.006933 |
| d:Bacteria;p:Actinobacteria;c:Actinobacteria;o:Gaiellales;f:Gaiellaceae;g:Gaiella | 6 | 4 | 0.007706 |
| d:Bacteria;p:Latescibacteria | 96 | 25 | 0.008641 |

Table S5 Enriched microbes with the fold change of OF/CF > 2 in the R2 system. Only the microbes with hypergeometric *P*-value < 0.01 are shown. (All assigned taxa: 13256 / 17683, FC > 2: 2336)

| Taxa (taxonomy) | OTUs with taxa | OF-enriched OTUs with taxa | *P*-value |
| --- | --- | --- | --- |
| d:Bacteria;p:Proteobacteria | 2470 | 578 | 2.86E-16 |
| d:Bacteria;p:Bacteroidetes | 1093 | 283 | 5.57E-13 |
| d:Bacteria;p:Acidobacteria;c:Acidobacteria_Gp6;g:Gp6 | 134 | 59 | 9.08E-13 |
| d:Bacteria;p:Bacteroidetes;c:Bacteroidetes_incertae_sedis | 76 | 39 | 2.14E-11 |
| d:Bacteria;p:Acidobacteria;c:Acidobacteria_Gp6 | 207 | 75 | 9.81E-11 |
| d:Bacteria;p:Proteobacteria;c:Gammaproteobacteria;o:Xanthomonadales | 54 | 28 | 1.07E-08 |
| d:Archaea;p:Thaumarchaeota | 57 | 24 | 1.32E-05 |
| d:Bacteria;p:Bacteroidetes;c:Sphingobacteriia;o:Sphingobacteriales;f:Chitinophagaceae | 172 | 53 | 1.53E-05 |
| d:Archaea;p:Thaumarchaeota;o:Nitrososphaerales;f:Nitrososphaeraceae | 22 | 13 | 1.55E-05 |
| d:Bacteria;p:Actinobacteria;c:Actinobacteria;o:Actinomycetales;f:Nocardioidaceae | 28 | 15 | 1.74E-05 |
| d:Bacteria;p:Proteobacteria;c:Betaproteobacteria | 318 | 85 | 2.79E-05 |
| d:Bacteria;p:Proteobacteria;c:Gammaproteobacteria;o:Xanthomonadales;f:Xanthomonadaceae | 42 | 19 | 3.11E-05 |
| d:Bacteria;p:Proteobacteria;c:Gammaproteobacteria | 287 | 78 | 3.17E-05 |
| d:Archaea;p:Thaumarchaeota;o:Nitrososphaerales | 27 | 14 | 5.37E-05 |
| d:Bacteria;p:Proteobacteria;c:Alphaproteobacteria;o:Sphingomonadales | 45 | 19 | 9.92E-05 |
| d:Bacteria;p:Bacteroidetes;c:Flavobacteriia | 70 | 25 | 0.000223 |
| d:Bacteria;p:Proteobacteria;c:Alphaproteobacteria | 483 | 114 | 0.000421 |
| d:Bacteria;p:Proteobacteria;c:Gammaproteobacteria;o:Xanthomonadales;f:Sinobacteraceae | 8 | 6 | 0.000602 |
| d:Bacteria;p:Actinobacteria;c:Actinobacteria;o:Gaiellales | 17 | 9 | 0.001003 |
| d:Bacteria;p:Verrucomicrobia | 579 | 131 | 0.00101 |
| d:Bacteria;p:Bacteroidetes;c:Sphingobacteriia;o:Sphingobacteriales | 236 | 60 | 0.001534 |
| d:Bacteria;p:Acidobacteria;c:Acidobacteria_Gp4;g:Gp4 | 40 | 15 | 0.002247 |
| d:Bacteria;p:Bacteroidetes;c:Flavobacteriia;o:Flavobacteriales;f:Flavobacteriaceae;g:Flavobacterium | 29 | 12 | 0.002329 |
| d:Bacteria;p:Actinobacteria;c:Actinobacteria | 347 | 82 | 0.002461 |
| d:Bacteria;p:Acidobacteria;c:Acidobacteria_Gp4 | 130 | 36 | 0.002822 |
| d:Bacteria;p:Bacteroidetes;c:Flavobacteriia;o:Flavobacteriales | 65 | 21 | 0.002922 |
| d:Bacteria;p:Bacteroidetes;c:Flavobacteriia;o:Flavobacteriales;f:Flavobacteriaceae | 53 | 18 | 0.003093 |
| d:Bacteria;p:Actinobacteria;c:Actinobacteria;o:Actinomycetales;f:Nocardioidaceae;g:Nocardioides | 10 | 6 | 0.003267 |
| d:Bacteria;p:Cyanobacteria/Chloroplast;c:Cyanobacteria;f:Family_I | 13 | 7 | 0.003327 |
| d:Bacteria;p:Proteobacteria;c:Alphaproteobacteria;o:Rhizobiales | 116 | 32 | 0.004989 |
| d:Bacteria;p:Proteobacteria;c:Deltaproteobacteria;o:Desulfuromonadales;f:Desulfuromonadaceae | 3 | 3 | 0.005467 |
| d:Bacteria;p:Cyanobacteria/Chloroplast;c:Cyanobacteria;f:Family_I;g:GpI | 3 | 3 | 0.005467 |
| d:Bacteria;p:Proteobacteria;c:Gammaproteobacteria;o:Xanthomonadales;f:Sinobacteraceae;g:Steroidobacter | 3 | 3 | 0.005467 |
| d:Bacteria;p:Acidobacteria;c:Acidobacteria_Gp5 | 28 | 11 | 0.005641 |
| d:Archaea;p:Thaumarchaeota;o:Nitrososphaerales;f:Nitrososphaeraceae;g:Nitrososphaera | 11 | 6 | 0.006121 |
| d:Bacteria;p:Acidobacteria | 1145 | 233 | 0.006981 |
| d:Bacteria;p:Latescibacteria | 96 | 27 | 0.007141 |

Table S6 Enriched microbes with the fold change of OF/CF < 0.5 in the R1 system. Only the microbes with hypergeometric *P*-value < 0.01 are shown. (All assigned taxa: 13256 / 17683, FC > 2: 1862)

| Taxa (taxonomy) | OTUs with taxa | OF-enriched OTUs with taxa | *P*-value |
| --- | --- | --- | --- |
| d:Bacteria;p:Proteobacteria;c:Deltaproteobacteria | 458 | 113 | 5.23E-10 |
| d:Bacteria;p:Cyanobacteria/Chloroplast;c:Cyanobacteria | 72 | 31 | 1.86E-09 |
| d:Bacteria;p:Cyanobacteria/Chloroplast | 147 | 44 | 4.61E-07 |
| d:Bacteria;p:Proteobacteria;c:Betaproteobacteria | 318 | 76 | 1.38E-06 |
| d:Bacteria;p:Proteobacteria | 2470 | 420 | 2.47E-06 |
| d:Bacteria;p:Verrucomicrobia;c:Subdivision3 | 262 | 64 | 4.31E-06 |
| d:Archaea;p:Euryarchaeota;c:Methanomicrobia | 28 | 12 | 0.000194 |
| d:Bacteria;p:Actinobacteria;c:Actinobacteria;o:Actinomycetales | 201 | 47 | 0.000235 |
| d:Bacteria;p:Bacteroidetes;c:Sphingobacteriia;o:Sphingobacteriales | 236 | 53 | 0.000287 |
| d:Bacteria;p:Actinobacteria;c:Actinobacteria;o:Actinomycetales;f:Mycobacteriaceae | 6 | 5 | 0.000288 |
| d:Bacteria;p:Verrucomicrobia;c:Spartobacteria | 50 | 17 | 0.000294 |
| d:Archaea;p:Euryarchaeota | 38 | 14 | 0.00039 |
| d:Bacteria;p:Chloroflexi | 240 | 53 | 0.000442 |
| d:Bacteria;p:Bacteroidetes;c:Sphingobacteriia;o:Sphingobacteriales;f:Chitinophagaceae | 172 | 40 | 0.000742 |
| d:Bacteria;p:Proteobacteria;c:Alphaproteobacteria;o:Sphingomonadales | 45 | 15 | 0.000835 |
| d:Bacteria;p:Proteobacteria;c:Deltaproteobacteria;o:Syntrophobacterales;f:Syntrophobacteraceae | 42 | 14 | 0.001234 |
| d:Bacteria;p:Proteobacteria;c:Deltaproteobacteria;o:Syntrophobacterales | 56 | 17 | 0.001278 |
| d:Bacteria;p:Proteobacteria;c:Alphaproteobacteria;o:Sphingomonadales;f:Sphingomonadaceae | 23 | 9 | 0.002653 |
| d:Bacteria;p:Actinobacteria;c:Actinobacteria;o:Actinomycetales;f:Streptomycetaceae | 12 | 6 | 0.003291 |
| d:Bacteria;p:Bacteroidetes;c:Sphingobacteriia | 304 | 60 | 0.003526 |
| d:Bacteria;p:Proteobacteria;c:Gammaproteobacteria;o:Xanthomonadales;f:Xanthomonadaceae | 42 | 13 | 0.003824 |
| d:Bacteria;p:Proteobacteria;c:Betaproteobacteria;o:Burkholderiales | 53 | 15 | 0.005075 |
| d:Bacteria;p:Proteobacteria;c:Deltaproteobacteria;o:Myxococcales | 100 | 24 | 0.005188 |
| d:Archaea;p:Euryarchaeota;c:Methanomicrobia;o:Methanosarcinales | 13 | 6 | 0.005391 |
| d:Bacteria;p:Cyanobacteria/Chloroplast;c:Cyanobacteria;f:Family_I | 13 | 6 | 0.005391 |
| d:Bacteria;p:Proteobacteria;c:Alphaproteobacteria | 483 | 88 | 0.005455 |
| d:Bacteria;p:Actinobacteria;c:Actinobacteria | 347 | 66 | 0.005642 |
| d:Bacteria;p:Planctomycetes;c:Planctomycetia;o:Planctomycetales | 59 | 16 | 0.006095 |
| d:Bacteria;p:Proteobacteria;c:Gammaproteobacteria;o:Xanthomonadales | 54 | 15 | 0.006133 |
| d:Bacteria;p:Planctomycetes;c:Planctomycetia;o:Planctomycetales;f:Planctomycetaceae | 45 | 13 | 0.007304 |
| d:Bacteria;p:Chloroflexi;c:Anaerolineae | 50 | 14 | 0.007389 |
| d:Bacteria;p:Chloroflexi;c:Anaerolineae;o:Anaerolineales | 22 | 8 | 0.007631 |
| d:Bacteria;p:Acidobacteria;c:Acidobacteria_Gp3 | 231 | 46 | 0.008272 |
| d:Bacteria;p:candidate_division_WPS-2 | 66 | 17 | 0.008318 |
| d:Bacteria;p:Chloroflexi;c:Anaerolineae;o:Anaerolineales;f:Anaerolineaceae | 14 | 6 | 0.008325 |

Table S7 Enriched microbes with the fold change of OF/CF < 0.5 in the R2 system. Only the microbes with hypergeometric *P*-value < 0.01 are shown. (All assigned taxa: 13256 / 17683, FC > 2: 1608)

| Taxa (taxonomy) | OTUs with taxa | OF-enriched OTUs with taxa | *P*-value |
| --- | --- | --- | --- |
| d:Bacteria;p:Proteobacteria | 2470 | 404 | 3.23E-12 |
| d:Bacteria;p:Verrucomicrobia | 579 | 126 | 1.77E-11 |
| d:Bacteria;p:Proteobacteria;c:Betaproteobacteria | 318 | 77 | 1.23E-09 |
| d:Bacteria;p:Proteobacteria;c:Deltaproteobacteria;o:Desulfuromonadales | 56 | 25 | 1.35E-09 |
| d:Bacteria;p:Verrucomicrobia;c:Subdivision3 | 262 | 67 | 1.36E-09 |
| d:Bacteria;p:Bacteroidetes;c:Sphingobacteriia;o:Sphingobacteriales;f:Chitinophagaceae | 172 | 50 | 1.71E-09 |
| d:Bacteria;p:Bacteroidetes;c:Sphingobacteriia | 304 | 71 | 2.70E-08 |
| d:Bacteria;p:Bacteroidetes;c:Sphingobacteriia;o:Sphingobacteriales | 236 | 59 | 3.16E-08 |
| d:Bacteria;p:Proteobacteria;c:Deltaproteobacteria;o:Desulfuromonadales;f:Geobacteraceae;g:Geobacter | 7 | 7 | 3.82E-07 |
| d:Bacteria;p:candidate_division_WPS-2 | 66 | 23 | 1.39E-06 |
| d:Bacteria;p:Proteobacteria;c:Alphaproteobacteria | 483 | 93 | 3.10E-06 |
| d:Bacteria;p:Verrucomicrobia;c:Subdivision3;g:Subdivision3_genera_incertae_sedis | 31 | 14 | 5.01E-06 |
| d:Bacteria;p:Proteobacteria;c:Deltaproteobacteria;o:Desulfuromonadales;f:Geobacteraceae | 14 | 9 | 6.29E-06 |
| d:Bacteria;p:Verrucomicrobia;c:Spartobacteria | 50 | 18 | 1.15E-05 |
| d:Bacteria;p:Proteobacteria;c:Alphaproteobacteria;o:Rhizobiales | 116 | 31 | 1.39E-05 |
| d:Bacteria;p:Bacteroidetes | 1093 | 178 | 1.42E-05 |
| d:Bacteria;p:Cyanobacteria/Chloroplast;c:Chloroplast | 27 | 12 | 2.93E-05 |
| d:Bacteria;p:Acidobacteria | 1145 | 180 | 9.76E-05 |
| d:Bacteria;p:Cyanobacteria/Chloroplast;c:Chloroplast;f:Chloroplast | 22 | 10 | 0.000109 |
| d:Bacteria;p:Proteobacteria;c:Betaproteobacteria;o:Neisseriales | 7 | 5 | 0.000444 |
| d:Bacteria;p:Proteobacteria;c:Betaproteobacteria;o:Neisseriales;f:Neisseriaceae | 7 | 5 | 0.000444 |
| d:Bacteria;p:Acidobacteria;c:Acidobacteria_Gp3 | 231 | 45 | 0.000818 |
| d:Bacteria;p:Proteobacteria;c:Betaproteobacteria;o:Rhodocyclales | 11 | 6 | 0.000847 |
| d:Bacteria;p:Proteobacteria;c:Betaproteobacteria;o:Rhodocyclales;f:Rhodocyclaceae | 11 | 6 | 0.000847 |
| d:Bacteria;p:Proteobacteria;c:Gammaproteobacteria;o:Legionellales | 28 | 10 | 0.001113 |
| d:Bacteria;p:Proteobacteria;c:Betaproteobacteria;o:Burkholderiales | 53 | 15 | 0.001187 |
| d:Bacteria;p:Proteobacteria;c:Deltaproteobacteria | 458 | 77 | 0.001699 |
| d:Bacteria;p:Bacteroidetes;c:Cytophagia;o:Cytophagales;f:Cytophagaceae;g:Cytophaga | 3 | 3 | 0.001782 |
| d:Bacteria;p:Bacteroidetes;c:Sphingobacteriia;o:Sphingobacteriales;f:Chitinophagaceae;g:Niastella | 3 | 3 | 0.001782 |
| d:Bacteria;p:Proteobacteria;c:Alphaproteobacteria;o:Rhizobiales;f:Xanthobacteraceae | 6 | 4 | 0.002642 |
| d:Bacteria;p:Actinobacteria;c:Actinobacteria;o:Actinomycetales;f:Mycobacteriaceae | 6 | 4 | 0.002642 |
| d:Bacteria;p:Proteobacteria;c:Gammaproteobacteria;o:Legionellales;f:Coxiellaceae | 18 | 7 | 0.003601 |
| d:Bacteria;p:Verrucomicrobia;c:Spartobacteria;g:Spartobacteria_genera_incertae_sedis | 18 | 7 | 0.003601 |
| d:Bacteria;p:Bacteroidetes;c:Sphingobacteriia;o:Sphingobacteriales;f:Chitinophagaceae;g:Chitinophaga | 10 | 5 | 0.003883 |
| d:Bacteria;p:Cyanobacteria/Chloroplast;c:Chloroplast;f:Chloroplast;g:Bacillariophyta | 11 | 5 | 0.006414 |
| d:Archaea;p:Thaumarchaeota | 57 | 14 | 0.006986 |
| d:Bacteria;p:Proteobacteria;c:Gammaproteobacteria | 287 | 49 | 0.008116 |

Table S8 The correlation of top 20 OTUs based on total read counts between AUDPC (r<=-0.8) in taxonomic level among six soil types.

| OTU_taxonomy | Combination of six soil types read counts | r_value* |
| --- | --- | --- |
| k__Bacteria, p__Proteobacteria, c__Betaproteobacteria, o__Burkholderiales | 1833 | -0.91268 |
| k__Bacteria | 1568 | -0.84969 |
| k__Bacteria | 955 | -0.84771 |
| k__Bacteria, p__Acidobacteria, c__Acidobacteria_Gp16, g__Gp16 | 782 | -0.83539 |
| k__Bacteria, p__Proteobacteria | 671 | -0.81289 |
| k__Bacteria, p__Actinobacteria, c__Actinobacteria, o__Actinomycetales, f__Streptomycetaceae, g__Streptomyces | 582 | -0.86801 |
| k__Bacteria, p__Acidobacteria, c__Acidobacteria_Gp16 | 513 | -0.81154 |
| k__Bacteria, p__Proteobacteria | 506 | -0.89644 |
| k__Bacteria, p__Acidobacteria, c__Acidobacteria_Gp6, g__Gp6 | 488 | -0.87063 |
| k__Bacteria | 453 | -0.83919 |
| k__Bacteria | 440 | -0.80036 |
| k__Bacteria, p__Proteobacteria, c__Deltaproteobacteria | 382 | -0.87548 |
| k__Bacteria, p__Acidobacteria, c__Acidobacteria_Gp6, g__Gp6 | 380 | -0.84949 |
| k__Bacteria | 370 | -0.82336 |
| k__Bacteria | 367 | -0.81575 |
| k__Bacteria, p__Proteobacteria | 365 | -0.94654 |
| k__Bacteria | 349 | -0.85967 |
| k__Bacteria, p__Acidobacteria | 316 | -0.81679 |
| k__Bacteria | 288 | -0.82824 |
| k__Bacteria, p__Actinobacteria | 280 | -0.9112 |

* r_value : simple Person correlation coefficient

Table S9 The correlation of top 20 OTUs based on total read counts between AUDPC (r>=0.8) in taxonomic level among six soil types.

| OTU_taxonomy | Combination of six soil types read counts | r_value* |
| --- | --- | --- |
| k__Archaea, p__Thaumarchaeota | 8510 | 0.83851 |
| k__Bacteria, p__Proteobacteria, c__Alphaproteobacteria, o__Sphingomonadales | 4675 | 0.839873 |
| k__Bacteria, p__Bacteroidetes, c__Sphingobacteriia, o__Sphingobacteriales, f__Chitinophagaceae | 3616 | 0.873449 |
| k__Bacteria, p__Proteobacteria, c__Betaproteobacteria, o__Burkholderiales, f__Oxalobacteraceae | 1969 | 0.899761 |
| k__Bacteria, p__Acidobacteria, c__Acidobacteria_Gp4 | 1904 | 0.875487 |
| k__Bacteria, p__Proteobacteria, c__Alphaproteobacteria | 1663 | 0.83077 |
| k__Bacteria | 1643 | 0.843404 |
| k__Bacteria, p__Bacteroidetes, c__Sphingobacteriia, o__Sphingobacteriales, f__Chitinophagaceae, g__Niastella | 1437 | 0.817429 |
| k__Bacteria, p__Bacteroidetes, c__Sphingobacteriia, o__Sphingobacteriales, f__Chitinophagaceae | 1334 | 0.833647 |
| k__Bacteria, p__Firmicutes, c__Bacilli, o__Bacillales, f__Bacillaceae_1 | 1323 | 0.865401 |
| k__Bacteria, p__Bacteroidetes, c__Sphingobacteriia, o__Sphingobacteriales, f__Chitinophagaceae, g__Niastella | 1201 | 0.892152 |
| k__Archaea, p__Thaumarchaeota | 1143 | 0.842475 |
| k__Bacteria, p__Proteobacteria, c__Deltaproteobacteria | 1086 | 0.810151 |
| k__Bacteria, p__Bacteroidetes, c__Sphingobacteriia | 1061 | 0.941826 |
| k__Bacteria, p__Acidobacteria, c__Acidobacteria_Gp4 | 992 | 0.871466 |
| k__Bacteria, p__Acidobacteria, c__Acidobacteria_Gp3 | 952 | 0.839851 |
| k__Bacteria, p__Bacteroidetes, c__Sphingobacteriia, o__Sphingobacteriales, f__Chitinophagaceae | 914 | 0.862787 |
| k__Bacteria, p__Bacteroidetes, c__Sphingobacteriia, o__Sphingobacteriales, f__Chitinophagaceae, g__Flavisolibacter | 898 | 0.878162 |
| k__Bacteria | 862 | 0.883394 |
| k__Bacteria, p__Proteobacteria, c__Deltaproteobacteria, o__Desulfuromonadales | 810 | 0.939353 |

* r_value : simple Person correlation coefficient
